# Exploring lactic acid bacteria from fresh fruits, vegetables, and edible flowers: from biodiversity valorisation to food applications

**DOI:** 10.1007/s11274-026-04872-7

**Published:** 2026-03-06

**Authors:** Ester Presutto, Vittorio Capozzi, Maria Lucia Valeria de Chiara, Djamel Drider, Giuseppe Spano, Mariagiovanna Fragasso

**Affiliations:** 1 Department of Agriculture Food Natural Science, and Engineering (DAFNE), University of Foggia, Foggia, 71122 Italy; 2 Institute of Sciences of Food Production (ISPA), National Research Council (CNR), c/o CS-DAT, Via Michele Protano, Foggia, 71121 Italy; 3 UMR Transfrontalière BioEcoAgro INRAE 1158, Université de Lille (ULille), Lille, 59000 France

**Keywords:** Fresh produce, Edible flowers, Fructophilic LAB (FLAB), Metabarcoding, Bioprotective cultures, Exopolysaccharides

## Abstract

Fresh plant matrices of food interest host complex microbial communities. Within these microbial ecosystems, biochemical and ecological interactions are relevant in contributing to food quality and safety. In particular, lactic acid bacteria (LAB) play key roles in fermentative processes, bioprotection, and bioactivity. Despite their well-recognised importance in food biotechnology, the ecological dynamics and metabolic versatility of LAB in plant-based environments are still poorly explored. Understanding their diversity and how they adapt to matrix-specific stressors is crucial for identifying new strains with distinctive technological and biofunctional traits. This review summarises a selection of recent studies on LAB associated with fresh plant-derived matrices (i.e., fresh fruits, vegetables, and edible flowers). Particular attention is given to culture-dependent and culture-independent approaches employed for their identification. The main technological and functional aspects are also examined, aiming to assess their properties of interest, including resistance to adverse environmental conditions and mechanisms of microbial interaction. Furthermore, the discussion addresses the main biotechnological applications of selected LAB, including the development of fermented plant-based foods and beverages, the design of probiotic cultures/biocontrol solutions, and the valorisation of plant by-products. To our knowledge, this is the first review to provide an integrated overview of genomic and ecological insights into LAB associated with fresh plant matrices. This approach would help to improve understanding of LAB adaptive dynamics and identify sustainable drivers of innovation in agro-food systems.

## Introduction

Regular consumption of plant-based foods is a key component of a balanced diet, providing essential intake of micronutrients (i.e., vitamins and minerals), antioxidants, and other bioactive phytochemicals relevant to human health (Ramos et al. 2013). The World Health Organization (WHO) recommends a minimum daily intake of 400 g of fruits and vegetables, evenly distributed across five servings per day (WHO/FAO 2003). In response to growing demand for practical and healthy solutions, the food industry has developed innovative products, including minimally processed fruits and vegetables (Mao et al. 2021). Additionally, fresh plant sources, including leaves, tubers, roots, and bulbs, provide an interesting natural ecological niche for microorganisms.

**Fig. 1:**
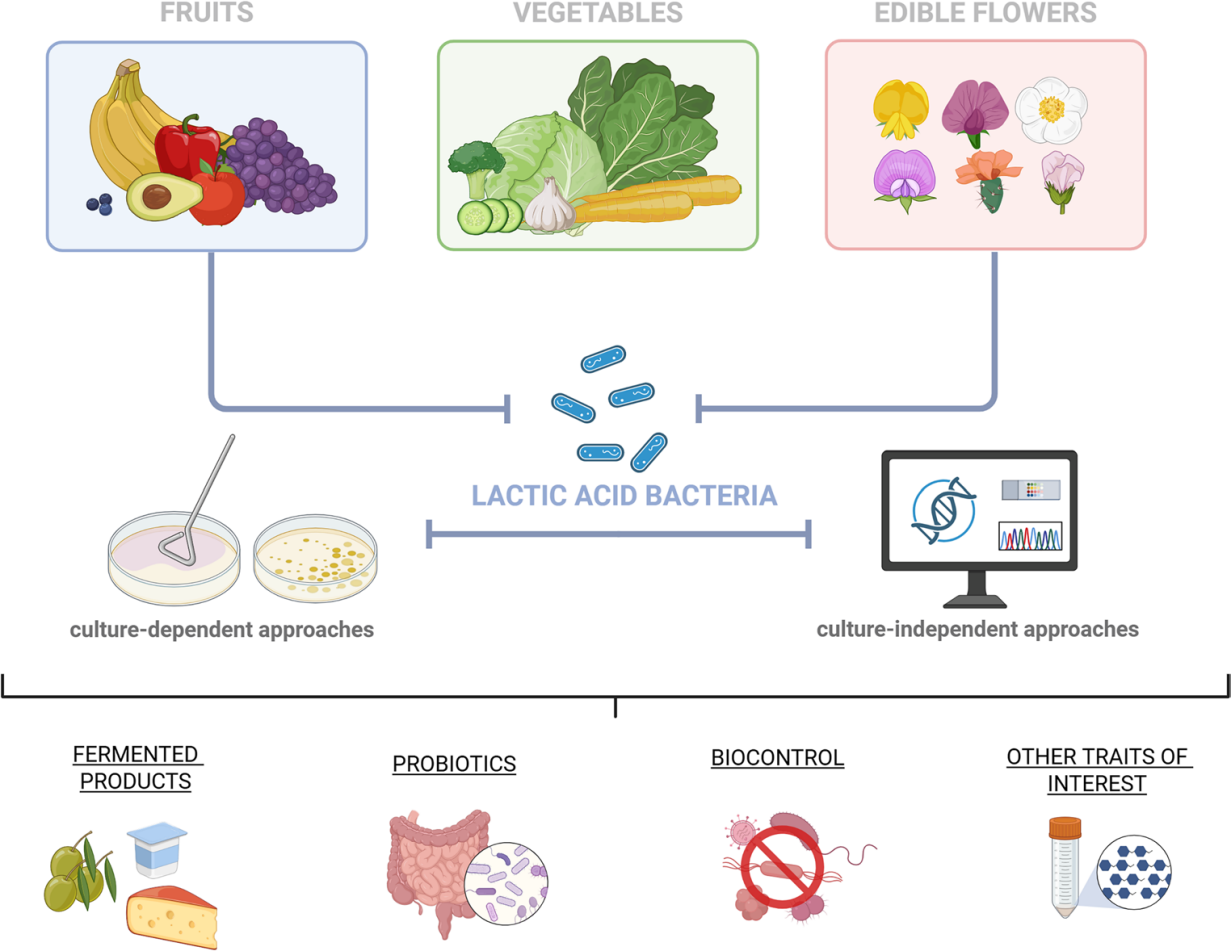
Workflow of LAB recovery from fruits, vegetables, and edible flowers through culture-based and culture-independent strategies, and their potential application in food biotechnology. Figure created in BioRender. Capozzi, V. (2026) https://BioRender.com/u6s3ypn

The structure of plant-associated microbial communities is strongly influenced by intrinsic (e.g., plant matrix, tissue physiology) and extrinsic factors (e.g., agronomic practices, climate, and post-harvest handling practices) (Olaimat and Holley 2012). Although yeasts and fungal species predominantly colonise such substrates, the presence of lactic acid bacteria (LAB) in fresh plant matrices is well documented (Di Cagno et al. 2013; Linares-Morales et al. 2018, 2020; Pimentel et al. 2021). LAB play a key role in food production, thanks to their traditional use as starter cultures, contributing to microbiological stability and the overall improvement of product quality (Arena et al. 2017). According to the definition of ‘food cultures’, some LAB strains can be deliberately introduced into the food chain as biocontrol agents, thanks to their ability to modulate the microbial ecosystem by synthesising metabolites with antimicrobial activity (Selmi et al. 2023; Cirat et al. 2024). This is particularly relevant because such cultures can improve food safety, reduce the use of chemical preservatives, and support the ecological transition in agri-food systems. At the same time, their strong ability to adapt to and colonise the gastrointestinal and urogenital tracts makes selected LAB strains among the most important probiotics used in medicine and veterinary practices (Liu et al. 2025; Tabashiri et al. 2025). Recent advances in comparative genomics and omics sciences have revealed the high genetic plasticity of LAB, clarifying their evolutionary basis and mechanisms of adaptation to various ecological niches and/or particularly hostile environments. The microbiodiversity of LAB represents a precious resource, and it is interesting to investigate the presence of specialisations within certain ecological niches and the behaviour of species with a demonstrated nomadic nature. From this perspective, fresh plant matrices represent heterogeneous ecosystems, in which associated microbial communities are exposed to temperature and humidity fluctuations, UV radiation, oxidative stress, and limited availability of fermentable nutrients (Filannino et al. 2018; Espinosa-Leal et al. 2022). Consequently, plant-associated LAB might have developed specific physiological and metabolic strategies that influence their phenotypic expression. For instance, some plant-derived LAB have been reported to metabolise plant sugars, detoxify phenolic compounds, and exhibit greater resilience to major abiotic and biotic factors (Yu et al. 2020). However, the limited representation of plant-derived LAB strains in international microbial biobanks highlights a clear knowledge gap that needs to be filled. Based on these premises, this review aims to emphasise the relevance of LAB from fresh plant matrices (i.e., fresh fruits and vegetables, and edible flowers) as an emerging source of microbial biodiversity. The objective is to outline the most recent evidence regarding their isolation and characterisation, examine the cultural and non-cultural approaches used for their identification, and discuss the main properties of biotechnological interest. Thus, the work aims to contribute to an integrated vision of the ecological and applicative potential of plant LAB, laying the foundations for their use in innovative, bio-inspired processes oriented towards sustainability (Fig. 1).

### Plant-associated microbiota in fresh edible matrices

Plants represent complex biological systems that coexist with diverse microorganisms that perform multiple physiological functions. Plant-associated microbiomes include epiphytic microorganisms, which colonise the surfaces of plant organs, and endophytes, which predominantly inhabit plant internal tissues. These microbial communities are continuously shaped by plant physiology and environmental fluctuations (Lindow and Brandl 2003; Saminathan et al. 2018). Their structure and function depend on multiple biotic (e.g., plant genotype, development stage, pollinator action, agronomic practices, interaction phenomena with other organisms and microorganisms), and abiotic factors (e.g., soil pH, soil type, organic matter content, salt concentration, possible presence of metals, climatic factors, humidity) (Kusstatscher et al. 2020; Vannette 2020; Santoyo 2022). Despite this high dynamism, some microbial taxa maintain a relatively stable composition and function over time. This conserved fraction constitutes the so-called *core* microbiome, the set of microorganisms consistently associated with the plant regardless of environmental variation. From a broader perspective, the plant and its microbiota can be considered a “meta-organism,” i.e., an integrated biological entity in which reciprocal interactions occur at the physiological, biochemical, and molecular levels (Yang et al. 2001; Berg et al. 2021). A holistic view introduces the concept of the ’plant holobiont’, where microorganisms and their host interact closely, exchanging signals and functions that support adaptation and overall plant performance (Sánchez-Cañizares et al. 2017). Increasing scientific relevance has been attributed to “dietary microbes”, defined as microorganisms naturally associated with food and regularly consumed as part of the human diet (Kwaasi 2003). Although the development of metabolically active microbial communities is traditionally associated with fermented foods, growing evidence demonstrates that fresh plant products also host diverse microbial consortia with desirable/beneficial effects (e.g., protechnological, biocontrol, probiotic, and plant-growth promoting functions), pathogens, or microorganisms involved in spoilage processes (Rezac et al. 2018; Gérard et al. 2024; Rincón and Neelam 2021; Abdelfattah et al. 2018). Therefore, understanding the microbial dynamics associated with fresh plant products intersects with issues of major scientific and social relevance, with implications ranging from food safety to the quality, shelf-life, and sustainability of fruit and vegetable production. According to Spurr (1994), the total microbial load of fresh fruits and vegetables ranges from 5.0 to 7.0 log CFU g^− 1^ with a predominance of yeasts and fungi. Several studies have examined the microbiome of the plant phyllosphere and the surface of fresh or minimally processed fruits, primarily to detect the presence of pathogenic microorganisms and assess their potential association with foodborne outbreaks (Lindow and Brandl 2003; Leff and Fierer 2013; Vermote et al. 2022). Environmental and agronomic variables, collectively defined as the ‘exposome’ (Wild 2005), deeply affect microbial community structure and the functional stability of the phytobiome. 16 S rRNA gene pyrosequencing revealed clear differences between organic and conventionally grown fruits and vegetables, with a prevalence of species belonging to the family *Enterobacteriaceae* within the plant tissues of conventionally cultivated products compared to the organic analogues (Leff and Fierer 2013). Consistent patterns were recently reported by Wicaksono et al. (2023), who observed higher bacterial diversity in naturally farmed apples and blueberries than in those produced using conventional horticultural systems. Similarly, ITS2 rDNA amplification revealed differences in the fungal microflora, depending on the cultivation method and sampling area (Abdelfattah et al. 2018). More recently, metabarcoding analysis (16 S and 18 S rRNA) revealed higher abundances of *Pantoea*, *Acinetobacter*, *Pseudomonas*, and *Ochrobactrum* in edible purple flowers (*Torenia fournieri* F. Lind.) grown under conventional agronomic practices, and a lower abundance of *Granulibacter* in those grown with biocompost (Santos de Morais et al. 2022). However, it is important to note that the literature in this field (i.e., the effect of farming systems on plant microbiota composition) is not unequivocal. While several metataxonomic studies report differences in microbial diversity between organic and conventional farming systems, other studies indicate that the cultivation system alone does not always significantly shape plant-associated microbiota, with plant species, tissue type, soil properties, and environmental conditions often being stronger drivers. In addition, increasing studies, grappling with managing complexity, rather than pointing to a single dominant factor, studied the role of multiple, often interconnected, biotic and abiotic variables, with limitations in the understanding of the single factor that exerts a prominent influence on the structure of plant-associated microbial communities (Berg et al. 2021; Chialva et al. 2022; Akponikpè et al. 2025). Despite the complexity of these ecosystems, it is interesting to highlight that LAB represent only a minor but functionally significant fraction of the plant microbiome. Species belonging to the genera *Enterococcus*, *Lactiplantibacillus*, *Lactobacillus*, *Lactococcus*, *Leuconostoc*, *Levilactobacillus*, and *Weissella* have been isolated from numerous plant tissues and on the surface of some tropical fruits (Ruiz Rodríguez et al. 2019; Yu et al. 2020). A relatively emerging subgroup within the LAB is represented by fructophilic lactic acid bacteria (FLAB). The main physiological characteristic that distinguishes this group is their marked preference for fructose metabolism, which explains their predominance in sugar-rich ecological niches, such as ripe fruits, floral nectar, and honey. FLAB also dominate the gut microbiota of nectar-feeding insects, where they contribute to sugar metabolism, modulation of microbial interactions, and maintenance of gut homeostasis. (De Simone et al. 2023). Their recurrent isolation from fruits and flowers (both whole and cut) through selective enrichment protocols suggests a probable transmission route via the bee digestive tract (Sakandar et al. 2019; Behare et al. 2020) (Table 1).

**Table 1:** LAB species isolated from different raw plant-derived products

Current name	Former name	Sources	References	
*Lactobacillus bulgaricus* *Lactobacillus helveticus* *Lactobacillus acidophilus* *Lactobacillus* sp.	*Lactobacillus bulgaricus* *Lactobacillus helveticus* *Lactobacillus. acidophilus* *Lactobacillus* sp.	Strawberry Beetroot juice Beetroot juice Cabbage, cauliflower, cluster bean, fenugreek, french beans, gherkins, ridged gourd, tomato	(Fevria and Hartanto 2019) (Zamanpour et al. 2023) (Zamanpour et al. 2023) (Junnarkar et al. 2019)	
*Lactiplantibacillus plantarum* *Lactiplantibacillus paraplantarum* *Lactiplantibacillus argentoratensis* *Lactiplantibacillus pentosus* *Lactobacillus* sp.	*Lactobacillus plantarum* *Lactobacillus paraplantarum* *Lactobacillus argentoratensis* *Lactobacillus pentosus* *Lactobacillus* sp.	Papaya, tomato, yellow pitaya, cherry tomato, apple, blackberry, blueberry, banana, pineapple, orange, *Açai* fruits, banyan tree, *Amrutha balli*, aloe, carob, strawberry tree fruits, mulberry, lettuce, arugula, broccoli florets, blueberry, taro plant leaves, cauliflower, beetroot, green peppers, cut flowers, date, grape, fig, lavender, guava fruit, olives Papaya and papaya plant leaves, cabbage Jackfruits *Üçburun* peppers Carrots, cucumber, radish	(Junnarkar et al. 2019; Bamidele et al. 2019; Samedi and Charles 2019; Nuhwa et al. 2019; Fessard and Remize 2019; Saguibo et al. 2019; Pinto et al. 2020a; Behare et al. 2020; Valencia-hernández et al. 2021; Abe Sato et al. 2021; Ngouénam et al. 2021; Li et al. 2021; Yang et al. 2022; Hou et al. 2023; Zamanpour et al. 2023; Vasundaradevi et al. 2024; Nuñez et al. 2024; Rocchetti et al. 2024; Cong et al. 2024; Güler 2024; Ouarabi et al. 2025; Foti et al. 2025) (Samedi and Charles 2019; Fessard and Remize 2019) (Pruthviraj et al. 2024) (Nalbant and Ersoy Omeroglu 2024) (Singh and Saini 2023)	
*Limosilactobacillus fermentum* *Lactobacillus* sp.	*Lactobacillus fermentum* *Lactobacillus* sp.	Cherry tomato, apple, blackberry, blueberry, cucumber, guava fruit Noni fruit	(Bamidele et al. 2019; Saguibo et al. 2019; Li et al. 2021) (Pruthviraj et al. 2023b)	
*Ligilactobacillus salivarius*	*Lactobacillus salivarius*	Beetroot juice	(Zamanpour et al. 2023)	
*Lentilactobacillus curieae* *Lentilactobacillus kosonis* *Lentilactobacillus buchneri*	*Lactobacillus curieae* *Lentilactobacillus kosonis* *Lactobacillus buchneri*	Artichoke tubers Artichoke tubers Blueberry	(Iraporda et al. 2024) (Iraporda et al. 2024) (Cong et al. 2024)	
*Latilactobacillus sakei* *Latilactobacillus curvatus*	*Lactobacillus sakei* *Lactobacillus curvatus*	Lychee, green peppers Red and green peppers, leaf peppers	(Nan et al. 2020; Nuñez et al. 2024) (Nuñez et al. 2024)	
*Lacticaseibacillus rhamnosus* *Lacticaseibacillus casei* *Lacticaseibacillus paracasei*	*Lactobacillus rhamnosus* *Lactobacillus casei* *Lactobacillus paracasei*	Fig, blueberry, *Üçburun* peppers Blueberry Blueberry, *Üçburun* peppers, lavender	(Ruiz Rodríguez et al. 2019; Nalbant and Ersoy Omeroglu 2024; Cong et al. 2024) (Cong et al. 2024) (Nalbant and Ersoy Omeroglu 2024; Cong et al. 2024; Güler 2024)	
*Companilactobacillus crustorum* *Companilactobacillus farciminis* *Companilactobacillus formosensis*	*Lactobacillus crustorum* *Lactobacillus farciminis* *Lactobacillus formosensis*	Lychee Lychee Lychee	(Nan et al. 2020) (Nan et al. 2020) (Nan et al. 2020)	
*Fructobacillus tropaeoli* *Fructobacillus durionis* *Fructobacillus pseudoficulneus* *Fructobacillus fructosus* *Fructobacillus* sp.	*Fructobacillus tropaeoli* *Fructobacillus durionis* *Fructobacillus pseudoficulneus* *Fructobacillus fructosus* *Fructobacillus* sp.	Papaya, fig, kaki Fig, kiwi Peach, banana *Narcissus*, sunflower, cut flowers Fresh *Kalparasa*	(Sakandar et al. 2019; Ruiz Rodríguez et al. 2019; Fessard and Remize 2019) (Sakandar et al. 2019; Ruiz Rodríguez et al. 2019) (Sakandar et al. 2019) (Sakandar et al. 2019; Saleh 2020; Behare et al. 2020; Khiabani et al. 2024) (Gopal et al. 2021)	
*Apilactobacillus kunkeei* *Apilactobacillus ozensis*	*Lactobacillus kunkeei* *Lactobacillus ozensis*	*Narcissus*, yellow rose, pink rose, different flowers Flowers	(Sakandar et al. 2019; Saleh 2020; Khiabani et al. 2024) (Khiabani et al. 2024)	
*Levilactobacillus brevis*	*Lactobacillus brevis*	Guava, custard apple flowers, apple, grapes, banana, orange blueberry, agave sap, green peppers, *Üçburun* peppers, flowers	(Ruiz Rodríguez et al. 2019; Saleh 2020; Rodrigues et al. 2021; Iga-Buitrón et al. 2023; Kodbal et al. 2024; Nalbant and Ersoy Omeroglu 2024; Nuñez et al. 2024; Cong et al. 2024)	
*Lactococcus lactis* *Lactococcus garvieae* *Lactococcus* sp.	*Lactococcus lactis* *Lactococcus garvieae* *Lactococcus* sp.	Papaya, fig, passion fruit flowers, medlar flowers, lychee, wild plant fruits, agave sap, *Üçburun* peppers, cut flowers Broccoli, cauliflowers Raw palm sap, green leafy vegetables	(Ruiz Rodríguez et al. 2019; Fessard and Remize 2019; Dinoto et al. 2020; Behare et al. 2020; Iga-Buitrón et al. 2023; Nalbant and Ersoy Omeroglu 2024) (Ibrahim et al. 2024) (Sequino et al. 2022; Ábrahám et al. 2024)	
*Leuconostoc pseudomesenteroides* *Leuconostoc mesenteroides* *Leuconostoc citreum* *Leuconostoc carnosum* *Leuconostoc* sp.	*Leuconostoc pseudomesenteroides* *Leuconostoc mesenteroides* *Leuconostoc citreum* *Leuconostoc carnosum* *Leuconostoc* sp.	Tomato, papaya, passion fruits, medlar flowers, kaki Papaya, passion fruits flowers, carrots, bell pepper, zucchini, cucumber, tangerine, guava, *pepino* fruits, broccoli, cauliflowers, red peppers, cut flowers Papaya, tomato, kaki, broccoli, cauliflowers Broccoli Fresh *Kalparasa*, raw palm sap, tomatoes	(Ruiz Rodríguez et al. 2019; Fessard and Remize 2019) (Ruiz Rodríguez et al. 2019; Fessard and Remize 2019; Linares-Morales et al. 2020; Behare et al. 2020; Schifano et al. 2021; Wang et al. 2023; Nuñez et al. 2024; Ibrahim et al. 2024; Khiabani et al. 2024) (Ruiz Rodríguez et al. 2019; Fessard and Remize 2019; Ibrahim et al. 2024) (Ibrahim et al. 2024) (Gopal et al. 2021; Sequino et al. 2022; Ábrahám et al. 2024)	
*Weissella cibaria* *Weissella confusa* *Weissella soli* *Weissella paramesenteroides* *Weissella viridescens* *Weissella oryzae* *Weissella* sp.	*Weissella cibaria* *Weissella confusa* *Weissella soli* *Weissella paramesenteroides* *Weissella viridescens* *Weissella oryzae* *Weissella* sp.	Tomato, passion fruit flowers, *naranjilla*, green peppers Papaya, cabbage, lettuce wild plant fruits Lychee and carrots Papaya, cassava and yam plant leaves, sapota, cherry, banana, orange, plum Lychee Wild plant fruits Pears	(Ruiz Rodríguez et al. 2019; Fessard and Remize 2019; Tenea et al. 2020; Nuñez et al. 2024) (Bamidele et al. 2019; Fessard and Remize 2019; Dinoto et al. 2020) (Schifano et al. 2021) (Samedi and Charles 2019; Fessard and Remize 2019; Pabari et al. 2020) (Nan et al. 2020) (Samedi and Charles 2019; Dinoto et al. 2020) (Sequino et al. 2022)	
*Periweissella fabalis*	*Weissella fabali*	Kaki	(Ruiz Rodríguez et al. 2019)	
*Enterococcus faecalis* *Enterococcus gallinarum* *Enterococcus casseliflavus* *Enterococcus hirae* *Enterococcus pseudoavium* *Enterococcus gilvus* *Enterococcus mundtii* *Enterococcus faecium* *Enterococcus durans* *Enterococcus lactis* *Enterococcus* sp.	*Enterococcus faecalis* *Enterococcus gallinarum* *Enterococcus casseliflavus* *Enterococcus hirae* *Enterococcus pseudoavium* *Enterococcus gilvus* *Enterococcus mundtii* *Enterococcus faecium* *Enterococcus durans* *Enterococcus lactis* *Enterococcus* sp.	Passion fruit flowers, wild plant fruits, different vegetable products, cut flower, tomatoes, mulberry, sugarcane plant leaves, hazel leaves Passion fruits flowers Passion fruit flowers, passion fruit, papaya, custard apple flowers, medlar flowers, green peppers Kaki, stevia Lychee Lychee Corn, Jalapeño pepper, green tomato, red apple, orange Bell pepper, cucumber, different vegetable products, blueberry, spoilt cabbage, flowers, lavender Flowers and different vegetable products Different vegetable products, flowers Fenugreek	(Ruiz Rodríguez et al. 2019; Samedi and Charles 2019; Dinoto et al. 2020; Linares-Morales et al. 2020; Nan et al. 2020; Alameri et al. 2022; Bal et al. 2024) (Ruiz Rodríguez et al. 2019) (Ruiz Rodríguez et al. 2019; Nuñez et al. 2024) (Ruiz Rodríguez et al. 2019; Saguibo et al. 2019) (Nan et al. 2020) (Nan et al. 2020) (Linares-Morales et al. 2020) (Saleh 2020; Linares-Morales et al. 2020; Alameri et al. 2022; Pruthviraj et al. 2023a; Olamide 2024; Güler 2024) (Nuhwa et al. 2019; Saleh 2020; Alameri et al. 2022) (Nuhwa et al. 2019; Alameri et al. 2022) (Junnarkar et al. 2019)	
*Pediococcus pentosaceus* *Pediococcus acidilactici*	*Pediococcus pentosaceus* *Pediococcus acidilactici*	*Mandacuru*, *Açai* fruits, cucumber, red peppers, spoilt cabbage, leek, parsley Different vegetable product, red peppers	(Bamidele et al. 2019; Saguibo et al. 2019; Abe Sato et al. 2021; Olamide 2024; Nuñez et al. 2024; de Vasconcelos Medeiros et al. 2024) (Alameri et al. 2022; Nuñez et al. 2024)	
*Streptococcus lutetiensis*	*Streptococcus lutetiensis*	Cape goose-berry fruit	(Saguibo et al. 2019)	

*Lactobacillus* (*Lb*.); *Lactiplantibacillus* (*Lpb*); *Limosilactobacillus* (*Lmb*); *Ligilactibacillus* (*Lgb*.); *Lentilactobacillus* (*Lnb*.); *Latilactobacillus* (*Ltb*.); *Lacticaseibacillus* (*Lcb*.); *Companilactobacillus* (*Cpb.*); *Fructobacillus* (*Fb*.); *Apilactobacillus* (*Apb*.); *Levilactobacillus* (*Lvb*.); *Lactococcus* (*Lc*.); *Leuconostoc* (*Leuc*.); *Weissella* (*W*.); *Periweissella* (*Pw*.); *Enterococcus* (*E*.); *Pediococcus* (*P*.); *Streptococcus* (*S*.)

### Culture-independent identification approaches

Taxonomic identification of LAB is a crucial aspect in assessing their biotechnological potential. While industrial and functional applications of LAB often depend on strain-specific characteristics, identification at the genus or species level is generally sufficient, especially when the balance of resolution, speed, and costs is considered (Temmerman et al. 2004). However, traditional culture-dependent methods tend to underestimate the real microbial diversity by excluding microorganisms that are difficult to cultivate in vitro (Saminathan et al. 2018). To address these challenges, several methods have been used to study the presence, abundance, and physiological status of LAB in fresh and dried foods (Table 2). Historically, quantitative PCR (qPCR), PCR-DGGE targeting 16 S rDNA regions, and BIOLOG metabolic profiling were successfully applied to monitor and study LAB communities in the phyllosphere (Zwielehner et al. 2008). Additional culture-independent methods (e.g., digital PCR, fluorescence in situ hybridisation (FISH), and flow cytometry) may offer valuable tools for assessing LAB presence and viability in more complex plant-derived matrices (El Sheikha 2018). Flow cytometry, particularly when combined with fluorescent probes or viability markers, enables highly sensitive discrimination between metabolically active and inactive cells, overcoming several limitations of conventional culture-based techniques (Chen et al., 2024). In recent years, continuous advances in genomics have expanded the analytical toolbox through high-throughput sequencing (HTS) platforms, including 16 S rRNA gene metabarcoding and shotgun metagenomics. These methods enable rapid, comprehensive characterisation of microbial biodiversity by parallel processing millions of DNA fragments, providing qualitative and quantitative insights into both dominant and low-abundance taxa (Ursell et al. 2012; Medina et al. 2016).

**Table 2:** Representative applications of culture-independent approaches for LAB detection in fresh plant-based matrices

LAB detected	Source of isolation	Molecular method used	Advantages	Limitations	Location of sample collection	References
*Lactobacillus* sp. and *Leuconostoc* sp.	Tomatoes, spinach, green olives, and dried figs	Shotgun metagenomic, Illumina MiSeq	High read depth; suitable for taxonomic profiling	High DNA quality is required, expensive, and computationally demanding	San Francisco, California	(Soto-Giron et al. 2021)
*Leuconostoc* sp., *Leuconostoc mesenteroides*, and *Fructobacillus*	Fresh *Kalparasa*	16 S rRNA gene-based metagenomic analysis (V3–V4 region), Illumina HiSeq	High sequencing depth and accuracy enable comprehensive profiling of dominant/low-abundance taxa	High cost, requires advanced bioinformatics tools, and large computational resources	India	(Gopal et al. 2021)
*Leuc. citreum*,* Leuc. pseudomesenteroides*,* E. casseliflavus*, *E. faecium*, *W. cibaria*,* W. bombi*,* Lc. lactis* subsp. *hordniae*, and *Lc. lactis* subsp. *lactis*	Fresh native fruits and flowers	Shotgun metagenomic, Ion Torrent Personal Genome Machine (PGM)	High resolution	Amplification bias cannot assess cell viability	Northern Argentina	(Vermote et al. 2022)
*Leuconostoc* sp. and *Lactococcus* sp.	Raw palm sap	Full-length 16 S rRNA metabarcoding, Oxford Nanopore Technology (ONT)	Portable platform; enables real-time; long-read sequencing	Lower accuracy than Illumina; requires careful data cleaning	Bangladesh	(Ábrahám et al. 2024)

#### 16S rRNA metabarcoding analyses

Metabarcoding is now a widely adopted next-generation sequencing (NGS) technique. Basically, a specific genomic region (e.g., a fragment of the 16 S rRNA gene for bacteria, or the 18 S rRNA gene or ITS region for fungi) is selectively amplified and sequenced using high-throughput technologies, allowing the identification of highly diverse microbial communities in food and environmental matrices (Leech et al. 2020). 16 S rRNA gene sequencing has been widely applied to profile bacterial composition at the genus level in various substrates, including palm sap, ready-to-eat salads, leeks, romaine lettuce, garlic, cabbage, and wildflowers (Yu et al. 2020; Mantegazza et al. 2022; Ábrahám et al. 2024). A study by Gopal et al. (2021) investigated the microbial dynamics of *Kalparasa*, a fresh, unfermented sap derived from the coconut palm, during spontaneous fermentation. Taxonomic profiling based on 16 S rRNA gene sequencing (V3–V4 region) revealed that the genus *Leuconostoc* predominated in the fresh product (61.15%). By the end of the fermentation process, the relative abundance of *Leuconostoc* decreased to 39.3%, while *Fructobacillus* accounted for 15.47% of the overall bacterial community, roughly twice the proportion observed in the corresponding unfermented sample (7.74%). Although 16 S rRNA gene amplification is useful for broad microbial profiling, it has significant limitations, especially when distinguishing closely related taxa at the species level is required. These limitations in taxonomic resolution are primarily due to current dependence on PCR, which can introduce biases related to primer specificity, differential amplification efficiency, and chimaera formation, potentially distorting estimates of relative abundances (Muñoz-Martinez et al. 2025). To overcome these challenges, Milani et al. (2018) developed a profiling approach based on amplification of the transcribed spacer sequence between 16 S and 23 S rRNA, combining genus-specific primers with a script for the QIIME software suite to identify lactobacilli at the phylotype level in highly complex samples. Fessard and Remize (2019) employed an approach based on the sequencing of the *recA* and *pheS* genes, combined with genotyping by rep-PCR using the (GTG)₅ primer, in order to discriminate strains at the intraspecific level.

With its strengths and limitations, metabarcoding offers a good compromise, particularly given its compatibility with most current sequencing platforms and technologies. However, when a broader characterisation is required, shotgun metagenomics may offer a more suitable untargeted strategy.

#### Shotgun metagenomics

Shotgun metagenomic sequencing represents one of the most powerful culture-independent strategies for analysing the complexity of microbial communities. This untargeted approach sequences the entire pool of DNA extracted from environmental or food matrices, thereby enabling comprehensive reconstruction of both taxonomic composition and functional gene repertoires. Unlike marker-based amplicon sequencing, which captures only a fraction of the genetic information, shotgun metagenomics provides strain-level resolution and enables direct inference of metabolic and ecological functions, including carbohydrate metabolism, stress response, and antimicrobial biosynthesis pathways (Quince et al. 2017). The earliest shotgun metagenomic evidence on the surface microbiota of fresh fruit dates back to 2022, when Vermote et al. (2022) explored the microbial composition of several tropical fruit and flower species collected in northern Argentina. The data revealed the predominance of LAB belonging to the genera *Enterococcus*, *Weissella*, and *Leuconostoc*, among other taxa. Despite the use of a robust combination of bioinformatics tools and reference databases, a significant fraction of the recovered sequences could not be assigned to known taxa, highlighting the current limitations in taxonomic resolution and database coverage for complex microbial environments. Indeed, despite its high analytical depth, shotgun metagenomics presents several operational limitations, including: (*i*) the high sequencing costs, (*ii*) the low sensitivity to low-abundance genomes, (*iii*) the inability to distinguish between live and dead cells, and (*iv*) the need for advanced bioinformatics expertise and significant computational resources to handle the large datasets generated (Wensel et al. 2022). The low intrinsic abundance of LAB in fresh plant matrices further complicates their detection using untargeted molecular techniques. This highlights the importance of adopting polyphasic strategies, in which culture-dependent methods are considered complementary and, in some cases, essential to high-throughput sequencing approaches. The optimal methodological framework cannot be defined in absolute terms, as it depends on variables that are difficult to compare directly, such as the desired taxonomic resolution, the availability of bioinformatics resources, and the nature of the matrix. Therefore, the choice of the most appropriate protocol should be tailored to the specific goals defined at the experimental design stage.

#### Multi-omics and functional inference

An expanding arsenal of multi-omics approaches has been increasingly adopted in fermented food systems to link community composition to functional output (Zhang et al. 2021; Lee et al. 2022; Butowski et al. 2025). In addition to genomic analyses previously described, key omics disciplines include transcriptomics (global analysis of gene transcripts), proteomics (global analysis of proteins), and metabolomics (global characterisation of metabolites). While metatranscriptomics provides a snapshot of active gene expression, metaproteomics and metabolomics, within the framework of microbial functionalisation and its potential translational application, allow the identification of proteins that are actually synthesised and the profiling of resulting metabolites and bioactive compounds, respectively (Xiong et al. 2024; Wu et al. 2025). Taken together, these high-throughput technologies represent a promising methodological breakthrough for the holistic understanding of complex biological systems. Considering as a target plant-associated LAB, over the past decade, several studies have employed silage as a methodological model to develop culture-independent strategies (Liu et al. 2026). From an ecological perspective, these matrices provide a suitable system for studying microbial communities in fermented plant-based environments, given their complex composition and the strong environmental selection imposed by fermentation. For example, the integrated analysis of culture-dependent and metagenomic data has clarified the microbial succession and identified key functional players involved in alfalfa silage fermentation (Wang et al. 2024). Another study assessed the response of the microbial community associated with corn stover undergoing fermentation guided by *Ltb. buchneri* PC-C1 and *Lpb. plantarum* PC1-1 (Okoye et al. 2023). Metabolomic analyses have shown that *Lpb. plantarum* MTD-1, inoculated with or without cellulase, was able to produce high levels of amino acids, peptides, and organic acid derivatives in response to a synergistic bacterium-enzyme mechanism in wheat straw silage (Du et al. 2025). In addition, an insightful review by Kahraman Ilıkkan et al. (2025) introduced the concept of “lactobacillomics” as a sort of dedicated subfield within the omics sciences, aiming to provide a comprehensive overview of this specific bacterial clade. In this light, the convergence of omics technologies within such a phylogenetically coherent group could represent a key turning point in modern biotechnology, especially given the extensive heterogeneity in adaptive mechanisms and strain-specific functionalities. While the increasing accessibility and standardisation of omics platforms may facilitate their application to fresh or minimally processed plant matrices, significant methodological challenges remain. These include the inherently low microbial density typically associated with LAB, the marked heterogeneity of plant-associated microbiota, and the difficulty in accurately detecting organisms that are not only present but also metabolically active and/or ecologically dominant. These constraints still largely contribute to confining the application of multi-omics approaches to silage/fermented food systems.

### Culture-dependent identification approaches

Bacterial growth and metabolic activity depend on the availability of a suitable biophysical and biochemical environment (e.g., nutrient supply, pH, redox potential). LAB are widely recognised as nutritionally demanding microorganisms that cannot generally proliferate on carbon-only minimal media (Filannino et al. 2019). Consequently, the selective isolation of certain LAB strains is often enhanced by targeted enrichment of culture media with micronutrients, organic compounds, and surfactants (e.g., Tween 80), or by adjusting the sugar content. Standard media such as de Man–Rogosa–Sharpe (MRS), used under anaerobic or microaerophilic conditions, are commonly employed for the recovery of strains belonging to more generalist and metabolically versatile species, such as *Leuconostoc* (*Leuc*.) *mesenteroides*, *Lactiplantibacillus* (*Lpb.*) *plantarum*, and *Weissella* (*W.*) *confusa* (De Man et al. 1960; Yang et al. 2022; Nuñez et al. 2024). An increasing number of studies have employed integrated phenotypic and molecular strategies to isolate and identify LAB from fresh fruits, vegetables, and edible flowers (Tables 3 and 4, and 5, respectively). From this point of view, it is of particular interest, both for ecological significance and biotechnological potential, to deepen the evidence relating to lactobacilli which constitute a taxonomically and functionally heterogeneous group, as evidenced by their widespread occurrence across highly diverse ecological niches. Recent advances in genomic sequencing and phylogenetic analyses have led to a comprehensive reorganisation of the genus *Lactobacillus* (*sensu lato*) into more taxonomically coherent and functionally specialised genera, including *Lactiplantibacillus*, *Lacticaseibacillus*, *Levilactobacillus*, *Limosilactobacillus*,* Companilactobacillus*,* Latilactobacillus*, and *Lentilactobacillus* (Zheng et al. 2020). This reclassification revealed substantial differences in gene repertoires, metabolic pathways, and adaptive strategies, emphasising the evolutionary specialisation and divergence of each new genus toward well-defined ecological niches and specific technological contexts (Lemos Junior et al. 2025). Among them, *Lpb. plantarum* stands out due to its metabolic flexibility and genomic plasticity. Such traits have enabled it to successfully colonise a broad spectrum of plant-associated habitats, including wild plants, flowers, and unconventional plant matrices, as well as tropical fruits (Table 2). Matrix-Assisted Laser Desorption/Ionisation Time-of-Flight Mass Spectrometry (MALDI-ToF MS) has proven particularly effective for rapid and reliable strain discrimination, especially when combined with 16 S rRNA gene analysis (Junnarkar et al. 2019; Rodrigues et al. 2021; Riolo et al. 2023). Additional research has also documented the presence of species such as *Lacticaseibacillus* (*Lcb.*) *rhamnosus*, *Lcb. casei*, and *Lcb. paracasei* in heterogeneous plant matrices, including figs, blueberries, and *Üçburun* peppers (Ruiz Rodríguez et al. 2019; Nalbant and Ersoy Omeroglu 2024; Cong et al. 2024). *Lvb. brevis* was shown to colonise highly differentiated ecological niches both nutritionally and structurally, including guava, custard apple flowers, agave sap, and orange (Ruiz Rodríguez et al. 2019; Rodrigues et al. 2021; Iga-Buitrón et al. 2023). This evidence is fully consistent with the recent comprehensive review by Lemos Junior et al. (2025), which describes *Levilactobacillus* as an acidophilus genus and associates *Lacticaseibacillus* with high genomic adaptability and technological versatility in plant fermentations. Species belonging to the genera *Leuconostoc* and *Weissella* have been found with comparable frequency. Indeed, their shared nutritional preference and overlapping biochemical profiles often complicate species delineation, as many *Weissella* strains exhibit phenotypic traits resembling those of *Leuconostoc* or heterofermentative lactobacilli. Consequently, phenotypic misclassifications between the two genera have historically led to several taxonomic re-evaluations (Fessard and Remize 2019). Tenea et al. (2020) employed *de novo* whole-genome sequencing and assembly services to characterise a novel strain belonging to the species *W. cibaria* isolated from the wild fruit of *naranjilla*. Less frequent species that were plausibly adapted to specific substrates included *Lactobacillus* (*Lb.*) *bulgaricus*, *Lb. helveticus*, *Lb. acidophilus*, *Enterococcus* (*E.*) *gilvus*, *E. hirae*, *E. pseudoavium*,* Streptococcus* (*S.*) *luteniensis*,* W. viridescens*, *W. soli*, *Periweissella* (*Pw.*) *fabalis*, *Ligilactibacillus* (*Lgb.*) *saliviarius*, and several species belonging to the genus *Companilactobacillus* (Table 2). These evolutionary losses reflect niche-specific selection pressures. The FLAB group constitutes a phylogenetically and functionally distinct clade within LAB, comprising exclusively members of the genera *Apilactobacillus* and *Fructobacillus*. FLAB species, which include both obligate fructophiles (e.g., *Apilactobacillus kunkeei*, *Apilactobacillus apinorum*, *Apilactobacillus micheneri*) and facultative ones (e.g., selected strains of *Lvb. brevis*), have undergone specific evolutionary adaptations that enable them to proliferate in fructose-rich niches such as floral nectar, ripe fruits, honey, and the gastrointestinal tract of pollinating insects. Indeed, these bacteria exhibit specific genomic adaptations, including reduced genomes, a lower guanine-cytosine (GC) content, partial loss of the *adhE* gene, and a requirement for external electron acceptors to regenerate NAD⁺, which is essential for their energy metabolism (Filannino et al. 2019; Konno et al. 2024). Recent studies have reported the presence of *Fructobacillus* (*Fb*.) *tropaeoli*, *Fb. durionis*, *Fb. fructosus*, and *Apilactobacillus* (*Apb.*) *kunkeei* in fresh and cut flowers, as well as in fructose-rich fruits such as papaya, figs, and kaki (Ruiz Rodríguez et al. 2019; Fessard and Remize 2019; Behare et al. 2020). Furthermore, Sakandar et al. (2019) detected the presence of *Fb. pseudoficulneus* in high-sugar matrices such as banana and peach, through cultures enriched with increasing concentrations (1–30%) of fructose. The evolution of FLAB represents a paradigmatic example of how ecological specialisation can drive adaptive and regressive evolution (Endo et al. 2018). Understanding this dynamic is crucial for selecting strains with desirable characteristics and for developing high-value biotechnological applications.

**Table 3:** Summary of LAB strains isolated from raw fruits, including matrix type, cultivation conditions, characterisation methods, and geographic origin

Autochthonous LAB species isolated	Source of isolation	Culture media and growth conditions	Characterisation (morphological, biochemical and molecular tests)	Location of sample collection	References
Putative *Lb. bulgaricus*	Strawberries	Nutrient Agar - MRS agar	Morphological characterisation by visual inspection, Gram stain	Aie Angek, Tanah Datar district, Indonesia	(Fevria and Hartanto 2019)
*Lmb. fermentum*, *Lpb. plantarum*, *S. lutetiensis*	Ripe guava, cape gooseberry fruit	Enrichment in MRS broth and incubation at 37° C for 1–2 days; plating in MRS agar (1% CaCO_3_) and incubation at 37° C for 48 h under anaerobic conditions	Gram stain and catalase test; 16 S rRNA gene amplification (universal primers: FC27 and RC1492)	Los Baños, Laguna, Philippines	(Saguibo et al. 2019)
*Fb. tropaeoli*, *Cb. paraalimentarius*/*kimchii*,* Lpb. paraplantarum*, *Lc. lactis*, *Leuc. pseudomesenteroides*, *Leuc. citreum*, *Leuc. mesenteroides*, *W. cibaria*, *W. confusa*, *W. paramesenteroides*, *W. soli*	Papaya and tomatoes	MRS agar + cicloesimide − 37° C for 72 h	Catalase test, identification by 16 S sequencing; pheS and recA genes sequenced if needed. PCR products sequenced by Sanger with specific primers. Intra-species typing by (GTG)₅ rep-PCR	Reunion Island, French overseas department	(Fessard and Remize 2019)
*Lvb. brevis*, *W. cibaria*, *Leuc. mesenteroides* subsp. *dextranicum/mesenteroides*, *Lc. lactis* subsp. *lactis*, *E. faecalis*, *E. gallinarum/casseliflavus*,* Fb. tropaeoli*,* Fb. durionis*,* Lc. lactis*,* Lcb. rhamnosus*,* Pw. fabalis*,* Leuc. pseudomesenteroides*,* Leuc. citreum*,* E. hirae*	Guaya, papaya, passion fruit, custard apple, medlar, mulberry, fig and khaki	MRS plates (LAB), fMRS + 2% fructose (fructophilic LAB). Enrichment: 5 mL FYP broth, 30 °C, 24 h. Refresh: 100 µL FYP broth, incubation. FYP agar + 0.5% CaCO_3_. Incubation 30 °C, 24–72 h (LAB, FLAB)	Molecular dereplication of isolates by rep-PCR genomic fingerprinting and sequencing of the V1 variable region of 16 S rRNA gene for representative isolates from different LAB and FLAB clusters	Tucumán, northern Argentina	(Ruiz Rodríguez et al. 2019)
*Fb. pseudoficulneus*,* Fb. durionis*	Apple, banana, Chinese peach, plum, melon, kiwi and lychee	FYP broth: 1% D-fructose, 0.5% polypeptone, 30 °C, 24 h, shaking. Inoculum: 50 µL sample in FYP broth with increasing fructose concentrations (up to 30%). Plating on FYP agar 30% fructose + 0.5% CaCO_3_. Further cultivation in GYP broth (glucose) at 30 °C, 24 h	16 S rRNA gene amplification and sequencing	Wuxi, (Jiangsu), China	(Sakandar et al. 2019)
*W. cibaria*	*Naranjilla*	Incubation: 10 g sample + sterile water, 5 days at room temperature; Inoculation and incubation: MRS agar, 37 °C for 24 h, anaerobic conditions	Gram stain, catalase, motility, indole tests; de novo NGS sequencing (Illumina HiSeq X Ten, Macrogen Inc.), PCR amplification	Sucumbios, Ecuador	(Tenea et al. 2020)
*Lc. lactis*,* Lc. garvieae*,* W. confusa*,* W. oryzae*, *E. faecalis*	Wild plant fruits	Direct sample on MRS agar, 10 days; 7 mL sterile cooking oil, reduced oxygen, anaerobic environment; dilution in 0.85% sterile saline, MRS + 1% CaCO_3_ plates, 37 °C for 24 h, anaerobic conditions	16 S rRNA gene amplification (universal primers: 27 F and 1492R) and sequencing	Tambrauw, West Papua, Indonesia,	(Dinoto et al. 2020)
*W. paramesenteroides*	Sapota, cherry, banana, orange and plum	Enrichment in MRS-BB broth with 0.5% sodium taurocholate and 0.5% galactooligosaccharides (or 0.5% fructooligosaccharides or xylooligosaccharides); 37 °C for 48 h, plating on MRS, 37 °C for 48 h	Gram stain and catalase test; 16 S rRNA gene amplification (universal primers: UNI 8 F and UNI 1492R) and sequencing	Local market, India	(Pabari et al. 2020)
*E. mundtii*, *Leuc. mesenteroides*	Green apple, red apple, peach, guava pear, green tomato, pomegranate orange, tangerine and grape	Enrichment of 1 mL in MRS broth, 26 °C for 18–24 h, anaerobic conditions; reseeding on MRS plates, 26 °C for 18 h	Gram stain, catalase test and evaluation of carbohydrate fermentation profiles. 16 S rRNA gene amplification and sequencing	Chihuahua City, Mexico	(Linares-Morales et al. 2020)
Putative and not identified LAB	Grape and banana	FYP (1% fructose) and fMRS (MRS + 1% fructose); incubation at 30 °C for 48 h, aerobic conditions; re-inoculation of selected colonies in respective culture broths, 30 °C for 48 h, aerobic conditions	16 S rRNA gene amplification and sequencing	Fermoy, Cork, Ireland	(Behare et al. 2020)
*Lpb. plantarum*	Yellow pitaya	MRS agar + aniline blue; colony incubation at 33 and 37 °C for 48 h, anaerobic conditions	16 S rRNA gene amplification and sequencing	Not reported	(Valencia-hernández et al. 2021)
*Lvb. brevis*,* Enterococcus* sp. (preliminary identification by MALDI-TOF); best probiotic candidates identified as *Lvb. brevis*	Apple, banana, grape and orange	MRS agar, 37 °C, 48 h, anaerobic conditions	Gram stain, catalase test and mobility evaluation, preliminary identification with MALDI-TOF, 16 S rRNA gene amplification (universal primers: 341 F and 806R) and sequencing	João Pessoa, Paraíba, Bràzil	(Rodrigues et al. 2021)
*Lpb. plantarum* and *Lmb. fermentum*	Cherry tomatoes, blueberries, blackberries, cherries and apples	Enrichment in MRS broth, 37 °C, 24 h, anaerobic, plating on MRS agar, 37 °C, 48 h, anaerobic conditions	Gram stain, catalase test, carbohydrate fermentation test, 16 S rRNA gene amplification and sequencing	Lishui, Nanjing and Nanjing Local Markets, China	(Li et al. 2021)
*Lpb. plantarum*	Banana, papaya, pineapple and orange	Plating on MRS agar, 30 °C, 48 h, anaerobic conditions	Gram stain, catalase test, CO_2_ production test, 16 S rRNA gene amplification and sequencing	Dschang, Menoua Division, Western Cameroon	(Ngouénam et al. 2021)
*P. pentosaceus*	*Mandacuru*	Enrichment in MRS broth, 37 °C, 72 h, 100 rpm; plating on MRS agar, 37 °C, 48 h, anaerobic conditions	Gram stain, catalase test. 16 S rRNA gene amplification and sequencing	Cuité, Pitombeira and Uiraúna (PB, Bràzil)	(de Vasconcelos Medeiros et al. 2024)
*Lpb. plantarum* and *P. pentosaceus*	*Açai* fruits	Plating on MRS agar, 37 °C, 48 h, anaerobic conditions	Gram stain, catalase test, cytochrome oxidase activity evaluation, 16 S rRNA gene amplification and sequencing	Combu Island, Belém-PA, Abaetetuba-PA, Breves-PA, Santarém-PA and Zé Doca-MA (Bràzil)	(Abe Sato et al. 2021)
*Lpb. plantarum*	Banana	Enrichment in MRS broth, 30 °C, 24 h, anaerobic, incubation of three concentration gradients on MRS + 0.5% CaCO_3_, 48 h, anaerobic conditions	Gram stain, catalase test, gas production from glucose, gelatin liquefaction test, lactic acid isomer determination, sugar fermentation (API 50 CH), 16 S rRNA gene amplification (universal primers: 27 F and 1492R) and sequencing	Wuzhishan Banana Park (Hainan, China)	(Yang et al. 2022)
*E. faecium*,* E. durans*,* E. lactis*,* P. acidilactici*	Tomato, strawberry and peach	MRS agar plating, anaerobic conditions, 37 °C, 48 h, CO₂ incubator, stock in 50% glycerol and 50% water.	Gram stain and catalase test; 16 S rRNA gene amplification (universal primers: 27 F and 1492R) and sequencing	Sharjah, United Arab Emirates	(Alameri et al. 2022)
*Limosilactobacillus* sp.	Noni fruit	Enrichment in MRS broth, 37 °C, 24 h, anaerobic, plating on MRS agar, 37 °C, anaerobic conditions	Morphological characterisation by visual inspection, Gram stain, catalase test, 16 S rRNA gene amplification (universal primers: 27 F and 1392R) and sequencing	Shivamogga, India	(Pruthviraj et al. 2023b)
*E. faecium*	Blue Cherry	Enrichment in MRS broth for 24 h at 30 °C and re-incubation at 37 °C for 24 h, anaerobic conditions	16 S rRNA gene amplification (universal primers: 27 F and 1392R) and sequencing	Mysuru, India	(Pruthviraj et al. 2023a)
*Leuc. mesenteroides*	*Pepino*	MRS + sucrose (5%) for 48 ore at 30° C, aerobic conditions (110 rpm)	Not clearly reported	Linfen, Shanxi, China	(Wang et al. 2023)
*Lpb. plantarum*	Banyan tree and *Amrutha balli*	Enrichment in MRS broth incubation in MRS agar at 37 °C for 48 h, anaerobic conditions	16 S rRNA gene amplification (universal primers: 8 F and 1391) and sequencing	Karnataka (Mysuru and Mandya districts), India	(Vasundaradevi et al. 2024)
*Lvb. brevis*	Banana	MRS agar and incubation at 37° C for 24–48 h, anaerobic conditions	Gram stain, catalase test, acid and gas production evaluation, 16 S rRNA gene amplification (universal primers: 16SrRNA-F and 16SrRNA-R)	Raichur, India	(Kodbal et al. 2024)
*Lpb. plantarum*	Samples of wild plants: medlar, aloe, carob, mulberry, and strawberry tree	MRS agar + 1.5% CaCO_3_; incubation at 37 °C for 48 h, aerobic conditions	16 S rRNA gene amplification with universal primer oligonucleotides (BSF8 and BSR1541)	Apulia, Italy	(Rocchetti et al. 2024)
*Lpb. argentoratensis*	Jackfruit	Enrichment in MRS broth, incubation in MRS agar at 37 °C for 24 h, plating on MRS at 37° C for 24 h	Gram stain, morphological evaluation and catalase test. 16 S rRNA gene amplification (universal primers: 27 F and 1392R) and sequencing	Shivamogga, India	(Pruthviraj et al. 2024)
*Lcb. rhamnosus*, *Lcb. paracasei*, *Lcb. casei*, *Lnb. buchneri*, *Lpb. planturum*, *Lvb. brevis*	Blueberry	MRS agar + CaCO_3_ (2%) and incubation at 37° C for 24–72 h, anaerobic conditions	Morphological characterisation by visual inspection, Gram stain and catalase test, 16 S rRNA gene amplification (universal primers: 27 F and 1492R)	Heihe City, Heilongjiang Province, China	(Cong et al. 2024)
*E. faecalis*	Tomatoes and white mulberry	Plating on FYP and incubation at 37° C for 24 h, aerobic conditions	Gram stain and catalase test. 16 S rRNA gene amplification (universal primers: 27 F and 1492R)	Kahramankazan, Ankara, Turkey	(Bal et al. 2024)
Not yet identified	Prickly pear, *ñangapirí*, *chañar*, *tutiá*	MRS agar	Gram stain, catalase test and sporulation evaluation;	*Gran Chaco*, South America, Argentina	(Rivas et al. 2025)

**Table 4:** Summary of LAB strains isolated from vegetables, including matrix type, cultivation conditions, characterisation methods, and geographic origin

Autochthonous LAB species isolated	Source of isolations	Culture media and growth conditions	Characterisation (morphological, biochemical, and molecular tests)	Location of sample collection	References
*Lpb. paraplantarum* *E. faecalis*, *Lpb. plantarum*,* W. paramesenteroides*	Papaya, yam, taro, sugar cane, and cassava leaves	Enrichment in MRS broth, incubation in MRS agar at 37 °C for 48 h, anaerobic conditions	Gram stain and catalase test, 16 S rRNA gene amplification (universal primers: 27 F and 1492R)	Pingtung, Taiwan	(Samedi and Charles 2019)
*P. pentosaceus*, *E. hirae* and other putative LAB not yet identified	Leek, stevia, and parsley	Enrichment in MRS broth and incubation at 37° C for 1–2 days; plating in MRS agar (1% CaCO_3_) and incubation at 37° C for 48 h, anaerobic conditions	Gram stain and catalase test; 16 S rRNA gene amplification (universal primers: FC27 and RC1492)	Los Baños, Laguna, Philippines	(Saguibo et al. 2019)
*Lpb. plantarum*, *Lactobacillus* sp., *Weissella.* sp., *Enterococcus* sp.	Cauliflower, gherkins, cluster beans, fenugreek, cow pea, bitter gourd, french beans, tomato, ridged and bottle gourd, and cucumber	Enrichment and incubation in MRS broth at 30° C for 48 h (microaerophilic conditions)	Metabolic analysis with Biolog, proteomic profiling with MALDI-TOF MS and 16 S rRNA gene sequencing	Junnar, Ambegaon and Khed, India	(Junnarkar et al. 2019)
*P. pentosaceus*, *W. confusa*, *Lpb. plantarum*	Cucumber, lettuce, and cabbage	Plating on MRS agar (conditions not crearly reported)	Gram stain, catalase and oxidase test, spore formation evaluation, 16 S rRNA gene amplification	Nigeria	(Bamidele et al. 2019)
*E. mundtii*, *E. faecium*, *Leuc. mesenteroides*	Chilaca pepper, jalapeño pepper, corn, courgette, lettuce, cucumber, pepper and soybean sprouts	Enrichment in MRS broth at 26° C for 18–24 h under anaerobic conditions and cultivation on MRS agar plates at 26 °C for 18 h	Gram stain, catalase test, evaluation of carbohydrate fermentation profiles, 16 S rRNA gene amplification (universal primers: 27 F and 1492R)	Chihuahua City, Mexico	(Linares-Morales et al. 2020)
LAB not identified	Cauliflower and spinach	FYP (1% fructose) and fMRS (MRS + 1% fructose) at 30° C for 48 h under aerobic conditions	16 S rRNA gene amplification	Fermoy, Cork, Ireland	(Behare et al. 2020)
*Lpb. plantarum*	Rocket and lettuce	MRS agar at 37° C for 48 h	16 S rRNA gene amplification (universal primers: 27 F and 1492R)	Porto, Portugal	(Pinto et al. 2020b)
*Leuc. mesenteroides*, *W. soli*	Carrots	Plating on MRS at 30° C for 24–48 h under anaerobic conditions	16 S rRNA gene amplification (universal primers: 8 F and 1492R)	Ortucchio, Abruzzo, Italy	(Schifano et al. 2021)
*E. faecium*,* E. durans*,* E. lactis*,* P. acidilactici*	Cucumber, lettuce, parsley and cabbage	MRS agar at 37° C for 24 h under anaerobic conditions	Gram stain and catalase test; 16 S rRNA gene amplification (universal primers: 27 F and 1492R)	Sharjah, United Arab Emirates	(Alameri et al. 2022)
*Lpb. plantarum*	Broccoli florets	Enrichment in MRS broth at 37° C for 24 h	Morphological characterisation by visual inspection, Gram stain, 16 S rRNA gene amplification (universal primers: 8 F and 16SR)	Dongguan, China	(Hou et al. 2023)
*Lpb. plantarum*,* Lb. helveticus*,* Lgb. salivarius*,* Lb. acidophilus*	Beetroot juice	Enrichment in MRS broth at 37° C for 24 h and cultivation on MRS agar plates at 37 °C for 48 h	Gram staining, catalase activity, CO_2_ production from glucose, evaluation of sugars fermentation (sucrose, mannitol, rhamnose, sorbitol and maltose), 16 S rRNA gene amplification with universal primers	Mashad, Iran	(Zamanpour et al. 2023)
Putative *Lpb. argentoratensis*	Carrot, radish and cucumber	Not reported	Morphological characterisation by visual inspection, Gram stain, 16 S rRNA gene amplification	Prayagraj, Allahabad, India	(Singh and Saini 2023)
*Lvb. brevis*, *Lc. lactis*	Agave sap	Isolation protocol not reported. Strain refreshment in MRS broth at 37 °C	Not reported	Mexico	(Iga-Buitrón et al. 2023)
*Leuc. mesenteroides* ssp. *mesenteroides*, *Leuc. mesenteroides* ssp. *dextranicum*, *Lc. garvieae*, *Leuc. citreum*, *Leuc. carnosum*	Cauliflower and broccoli	Enrichment in MRS broth at 30° C for 48 h and cultivation on MRS agar plates	Gram stain, identification confirmed with VITEK^®^2 system	Baghdad markets, Iraq	(Ibrahim et al. 2024)
*Ltb. curvatus*, *Lpb. plantarum*, *E. casseliflavus*, *P. acidilactici*, *Leuc. mesenteroides*, *Lvb. brevis*, *W. cibaria*, *P. pentosaceus*, *Ltb. sakei*	Red and green peppers	Cultivation on MRS agar at 37° C for 48 h under anaerobic/microaerophilic conditions	Microscopic morphological observation, Gram stain, catalase test. Characterisation at strain level by rep-PCR with specific primers (GTG)5	Tucumán, northern Argentina	(Nuñez et al. 2024)
*Lcb paracasei* ssp. *paracasei*, *Lc. lactis* ssp. *lactis*, *Lvb. brevis*, *Lcb. rhamnosus*, *Lpb. pentosus*	*Üçburun* peppers	Cultivation on MRS at *i*) 37 °C for 24 h (aerobic and anaerobic conditions) and *ii*) 30° C for 72 h under aerobic conditions	Gram staim, catalase test, oxidase test, CO_2_ production test, evaluation of carbohydrate fermentation profiles (kit API 50 CH), growth evaluation on M17, BHI, NA, MRS, identification by 50 CH API software	Turkey	(Nalbant and Ersoy Omeroglu 2024)
*Lnb kosonis*, *Lnb. curieae*	Jerusalem artichoke	Enrichment in FYP or IYP at 37 °C for 72 h and incubation at 37° C under aerobic conditions	Microscopic morphological observation, Gram stain and catalase and oxidase test, 16 S rRNA amplification	Jerusalem, Israel	(Iraporda et al. 2024)
*Lpb. plantarum*	Olive skin	Cultivation on MRS agar	Microscopic morphological observation, Gram stain, catalase, and sugar fermentation (API 50 CHL) tests, 16 S rRNA gene amplification (universal primers: 27 F and 1490R)	Catania, Italy	(Foti et al. 2025)

**Table 5:** Summary of LAB strains isolated from edible flowers, including matrix type, cultivation conditions, characterisation methods, and geographic origin

Autochthonous LAB species isolated	Edible flowers	Culture media and growth conditions	Characterisation (morphological, biochemical, and molecular tests)	Location of sample collection	References
*Fb. fructosus*,* Apb. kunkeei*	Narcissus, pink rose, red rose, yellow rose and sunflower	FYP + fructose (1% − 30%) at 30° C for 24 h	Morphological observation 16 S rRNA amplification, evaluation of carbohydrate fermentation profiles (kit API 50 CH)	Wuxi, (Jiangsu), China	(Sakandar et al. 2019)
*E. durans*, *Lc. lactis*, *Enterococcus* sp., *E. lactis*, *Lpb. plantarum*	*Gardenia jasminoides*, *Hibiscus syriacus*, *Solanum torvum*, *Leucaena leucocephala*	Enrichment of samples in MRS broth and incubation at 37 °C for 48–72 h under anaerobic conditions	Gram staining, catalase test and qualitative morphological identification; 16 S rRNA amplification	Nakhon Si Thammarat, Thailand	(Nuhwa et al. 2019)
*Lvb. brevis*,* W. cibaria*,* Leuc. mesenteroides* subsp. *mesenteroides*,* Leuc. pseudomesenteroides*,* Lc. lactis* subsp. *lactis*,* E. faecalis*,* E. gallinarum/casseliflavus*,* E. casseliflavus*,* Fb. tropaeoli*,* Fb. durionis*,* Lc. lactis*,* Leuc. citreum*,* E. hirae*	Papaya flowers, passion fruit flowers, meddler flowers, custard apple flowers	MRS plates (LAB), fMRS + 2% fructose (fructophilic LAB). Enrichment: 5 mL FYP broth, 30 °C, 24 h. Refresh: 100 µL FYP broth, incubation. FYP agar + 0.5% CaCO3. Incubation 30 °C, 24–72 h (LAB, FLAB)	Molecular dereplication of isolates by rep-PCR genomic fingerprinting and sequencing of the V1 variable region of 16 S rRNA gene for representative isolates from different LAB and FLAB clusters	Tucumán, northern Argentina	(Ruiz Rodríguez et al. 2019)
*Leuc. mesenteroides*, *Leuc. mesenteroides* subsp. *jonggajibkimchii*,* Fb. fructosus*,* Lc. lactis*, *E. faecalis*,* Lpb. plantarum*	Cut flowers	Cultivation in FYP (1% fructose) and fMRS (MRS + 1% fructose); incubation at 30 °C, 48 h in aerobiosis; colony transfer to respective broth media; incubation 30 °C, 48 h under aerobiosis	16 S rRNA gene amplification and sequencing	Kilworth, County Cork, Ireland	(Behare et al. 2020)
Unidentified coccoid LAB	Nopal flower	Enrichment in MRS broth; incubation 26 °C, 18–24 h, under anaerobic conditions; subculture on MRS agar; incubation 26 °C, 18 h	Gram stain, catalase test, evaluation of sugars fermentation 16 S rRNA gene amplification (universal primers: 27 F and 1492R) and sequencing	Chihuahua City, Mexico	(Linares-Morales et al. 2020)
*Fb. fructosus*, *Apb. kunkeei*,* E. durans*, *E. faecium*, *Lvb. brevis*	*Hibiscus rosa-sinensis*,* Rosa rugosa*,* Tagetes erecta*	Enrichment in MRS broth, 24 h, 30 °C; pellet recovery, resuspension in MRS broth; plating on MRS agar; incubation 30 °C, 72 h	16 S rRNA gene amplification (universal primers: 27 F and 1492R) and sequencing	Al-Jadriya, Baghdad, Iraq	(Saleh 2020)
*Lpb. plantarum*	Banana flowers	Enrichment in MRS broth, 24 h, 30 °C, anaerobic; incubation of three concentration gradients on MRS + 0.5% CaCO₃, 48 h, anaerobic	Gram stain, catalase test, gas production from glucose, gelatin liquefaction test, lactic acid isomer determination, sugar fermentation (API 50 CH), 16 S rRNA gene amplification (universal primers: 27 F and 1492R) and sequencing	Wuzhishan Banana Park (Hainan, China)	(Yang et al. 2022)
*E. faecium*,* Lpb. plantarum*,* Lcb. paracasei*	Lavender rhizosphere	Cultivation on Nutrient Agar and incubation at 28° C	Gram staining, bacterial identification by MALDI-TOF MS; protein profile acquisition; spectral comparison with MALDI Biotyper CA database for genus/species-level identification	Ankara, Turkey	(Güler 2024)

### LAB associated with fresh vegetables, fruit and edible flowers: from ecological significance to biotechnological applications in food fermentation and functionalisation

The importance of LAB, which constitute only a small portion (generally between 2.0 and 4.0 log CFU g^− 1^) of the indigenous microbiota of raw fruits and vegetables, is closely linked to their biotechnological potential. This growing interest makes them particularly relevant when recovered from raw fruits and vegetables, which are often exposed to human handling and are generally consumed without further processing (Ruiz Rodríguez et al. 2019). From an ecological perspective, these matrices are acidic, protein-poor, and sugar-rich environments (Naeem et al. 2012; Ouarabi et al. 2025). A summary of the main LAB species isolated from fresh fruits, vegetables, and edible flowers is reported in Table 2. The time frame considered for the bibliography (2019–2025) reflects the growing scientific attention to the exploration of unconventional and sustainable sources of new LAB strains with biotechnological relevance. LAB have long been used in food preservation due to their ability to extend the shelf life of fresh and highly perishable food products through lactic fermentation. This biological process combines the concept of safety with the optimisation of overall product quality, including technological, nutritional, functional, and sensory aspects, with important application implications (Capozzi et al. 2017; Ağagündüz et al. 2022). Over time, the introduction of selected starter cultures has redefined fermentation as a controlled and standardised biotechnological approach, ensuring greater process reproducibility and microbiological safety compared to spontaneous fermentation or traditional ‘back slopping’ (i.e., the reinoculation of a small aliquot of the previously fermented product) (Bintsis 2018). Fruits and vegetables are particularly suitable for lactic fermentation due to their high content of simple carbohydrates, polyphenols, vitamins, and dietary fibres. While earlier approaches traditionally focused on sensory aspects, the increasing demand for foods with high nutritional and functional value has progressively reoriented the concept of fermentation. This evolution marks a conceptual shift from the traditional view of fermented foods as inherently healthy, towards scientifically guided bioprocesses designed to maximise specific biofunctional outcomes (Abedin et al. 2024). A significant contribution in this direction comes from the study by Li et al. (2021), in which several *Lpb. plantarum* strains, previously isolated from fermented fruit-based substrates (apple, cherry, blackberry, and blueberry), were applied to blueberry juice fermentation. Among these, strains LSJ-TY-HYB-T7 and LSJ-TY-HYB-L16 showed strong viability (10 logCFU mL^− 1^ after 48 h) and exhibited a marked metabolic activity. This corresponded to reduced caffeic acid levels, increased phenolic content, and significant improvements in antioxidant indices (46.3% and 39.9% for strain T7, and up to 107.9% for L16). Beyond single-strain fermentation, a recent experimental study explored the effects of co-fermentation with *Lvb. brevis* and *Lc. lactis* on broccoli, a food known for its high content of compounds associated with antioxidant, antitumor, and immunosuppressive effects (i.e., glucosinolates, isothiocyanates, indoles, and flavonoids). In this case, mixed fermentation enabled the preservation of the main bioactive compounds, presenting an interesting opportunity for the development of functional foods from raw-use vegetables (Iga-Buitrón et al. 2023). Khiabani et al. (2024) evaluated the feasibility of LAB isolated from edible flowers for application in small-scale fermentation processes. The selected strains exhibited a pronounced acidifying capacity during the fermentation of oat-based beverages, lowering the pH to values between 3 and 4. In contrast, the acidifying capacity on soy- and almond-based matrices was limited to the species *Leuc. mesenteroides*, *Lc. lactis*, *W. paramesenteroides*, *W. minor*, *Fb. tropaeoli*, *W. bombi*, and *Apb. kunkeei*. A plausible explanation for this divergence in fermentation performance may lie in species-specific adaptive capacities and matrix-specific constraints, including differences in carbohydrate composition, buffering capacity, and the presence of inhibitory compounds.

Besides their fermentative role, several plant-associated LAB have shown bioprotective, probiotic, and other functional properties of interest. Table 6 provides representative cases that introduce the applications discussed in the following sections.

**Table 6:** Selected studies on potential food and biotechnological applications of LAB strains isolated from fresh fruits, vegetables, and edible flowers

LAB strains	Source of isolation	Properties details	Potential application	References	
*W. cibaria* UTNGt21O	*Naranjilla*	Bacteriocin-like peptide with inhibitory activity against *Salmonella enterica* and *Escherichia coli*; enhanced effect with EDTA	Antimicrobial formulations	(Tenea et al. 2020)	
*W. paramesenteroides* FX5 and FX9	Tropical fruits	Acid tolerance, prebiotic metabolism (GOS/FOS), inhibition of *Escherichia coli* and *Staphylococcus aureus*	Probiotic cultures	(Pabari et al. 2020)	
*Lpb. plantarum* 1B9 and 3A5	Banana and pineapple	High lactic acid production from fruit by-products	By-product valorisation	(Ngouénam et al. 2021)	
*Lpb. plantarum* LSJ-TY-HYB-T9*/*T7 and *Lmb. fermentum* SJ-TY-HYB-C22/L16	Cherry tomatoes, blueberries, blackberries, cherries, and apples	Increased phenolics and antioxidant capacity in fermented juice	Functional fermentation	(Li et al. 2021)	
*Lpb. plantarum* 10 A, 11 A, CG56, CZ97, CZ103, UFG 121, and NC8	Medlar flowers, aloe, carob, mulberry and strawberry tree	GI survival, immunomodulation, adhesion; pectin degradation	Probiotic/postbiotic potential	(Rocchetti et al. 2024)	
*Leuc. mesenteroides* RSG7	*Pepino*	Dextran production and improvement of structural properties in sucrose-rich foods	EPS production, food structuring	(Wang et al. 2023)	
*Leuc. mesenteroides* and *Lpb. plantarum*	Carrots epidermis	GI tolerance, pathogen inhibition, pro-longevity in *Caenorhabditis elegans*	Probiotic + systemic functionality	(Schifano et al. 2021; Pompa et al. 2023)	
*P. pentosaceus* C6	Spoilt cabbage	Tolerant to acidic pH (3 h) and 0.3% bile; showed highest growth rate at 72 h fermentation	Probiotic fermentation of cabbage juice	(Olamide 2024)	
*Lnb. kosonis* and *Lnb. curieae*	Jerusalem artichoke tubers	Acid and GI tolerance (> 88% survival); strong autoaggregation (61–81%); antagonism vs. *Escherichia coli* and *Bacillus cereus*; downregulation of proinflammatory response	Probiotic cultures	(Iraporda et al. 2024)	
*Lvb. brevis* 3M1 and *Lc. lactis* 3M8	Agave sap	Increased antioxidant activity (Caco-2); antiproliferative effect (HT29, HCT116); anti-inflammatory response; optimal at 600 µg/mL (day 6)	Functional food/CRC chemoprevention		(Iga-Buitrón et al. 2023)
*Lpb. plantarum* CG1	Banana fruits and flowers	Improved silage quality; tannin reduction	Silage fermentation	(Yang et al. 2022)	
*E. durans* FM12-1, *E. durans* FM12-2, *E. lactis* FM11-2, and *Lpb. plantarum* FM13-1	Edible flowers	Cholesterol assimilation, bile salt hydrolysis, and bile tolerance	Probiotic cultures	(Nuhwa et al. 2019)	
*E. durans*, *Apb. kunkeei*, *Fb. fructosus*, and *Lvb. brevis*	Edible flowers	In vitro antagonistic activity against *S. aureus* and *Pseudomonas aeruginosa*	Biocontrol cultures	(Saleh 2020)	
*E. faecium* PIM4, *E. mundtii* ELO8, TOV9 and JAV15, *Leuc. mesenteroides* PIM5, and CAL14	Bell pepper, corn, green tomato, jalapeño and zucchini	In vitro antagonistic activity against *L. monocytogenes* and *Fusarium oxysporum*	Biocontrol cultures	(Linares-Morales et al. 2020)	
*Lpb. plantarum* CM-3	Strawberry	In vitro and in vivo antifungal evaluation against *Botrytis cinerea*	Biocontrol cultures	(Chen et al. 2020)	
*Leuc. mesenteroides*,* Fb. fructosus*,* Apb. kunkeei*, and *Apb.* *ozensis*	Edible flowers	Glucan, fructan and galactan production, improvement of some of the technological properties in almond, oat and soya milk	Potential plant-based beverages applications	(Khiabani et al. 2024)	
*Lpb*. *plantarum* S-811	Cactus pears	Probiotic fermentation of fruit juice	Protechnological use	(Verón et al. 2023)	
*Lpb. plantarum* MYSVB1	Banyan tree fruit	GI tolerance, inhibitory effect against *Alternaria alternata*	Dual probiotic-antifungal use	(Vasundaradevi et al. 2024)	

### Bioprotection and antagonist activity

Under suitable growth conditions, LAB can produce a wide range of bioactive compounds with antimicrobial properties. These include organic acids (e.g., lactic and acetic acid), volatile compounds, and more complex secondary metabolites synthesised through specific biosynthetic pathways or generated via the bioconversion of extracellular substrates. These compounds differ in their modes of action and biological targets. Organic acids and volatile compounds primarily act by lowering pH and disrupting membrane integrity, creating unfavourable conditions for spoilage organisms. Bacteriocins (ribosomally synthesised antimicrobial peptides) often exert a more targeted mechanism, such as pore formation in microbial membranes or interference with essential enzymatic functions (Hernández-González et al. 2021). A detailed classification and mechanistic overview of LAB-derived antimicrobial compounds from diverse ecological environments, along with their inhibitory spectra, is provided in the recent review by Siedler et al. (2019). Here, we provide a targeted evaluation of antimicrobial metabolites from fresh plant-associated LAB, based on some representative in vitro and in vivo models. A membrane permeability assay showed that the enterolysin A-like bacteriocin produced by *W. cibaria* UTNGt21O inhibited *S. enterica* ATCC 51,741 (3200 AU mL^− 1^) and *E. coli* ATCC 25,922 (6400 AU mL^− 1^). The inhibitory effect followed a dose-time-dependent pattern, with a marked bacteriolytic response after 6 h of co-inoculation with 20 mM EDTA (Tenea et al. 2020). Recognising the role of such antagonistic metabolites is particularly relevant, considering the vulnerability of fresh fruit and vegetable products to microbial infections during the post-harvest stage, where microbiological spoilage significantly compromises both quality and yield. One of the major challenges in fresh produce preservation is contamination by *Botrytis cinerea*, a model fungal pathogen for soft-rot spoilage of fruits, known for its high spoilage potential and significant post-harvest economic impact (De Simone et al. 2020). Chen et al. (2020) reported that *Lpb. plantarum* CM-3 reduced the incidence and severity of grey mould on strawberries infected with *B. cinerea* by up to 75% compared with the untreated control after six days at 20 °C. These insights reinforce the role of LAB-derived bioactives as innovative, sustainable tools for developing bioprotective cultures tailored to post-harvest fruit preservation.

### Probiotic and postbiotic traits

In an integrated perspective between modulation and matrix preservation, some plant-derived strains are characterised by documented probiotic and postbiotic properties, with immunomodulatory and metabolic effects already observed in both in vitro and in vivo models. Strains belonging to *E. durans*, *E. lactis*, and *Lpb. plantarum* isolated from floral and plant matrices have shown good survival under simulated gastrointestinal conditions, cholesterol assimilation capacity, and a relevant impact on immune modulation in intestinal epithelium models (Nuhwa et al. 2019; Rocchetti et al. 2024). On Caco-2 epithelial cell lines, *Lvb. brevis* 3M1 and *Lc. lactis* 3M8 strains reduced the flagellin-induced inflammatory response, as determined by in vitro assays, suggesting potential use for restoring intestinal balance in inflammatory conditions (Iraporda et al. 2024). Comparable evidence was reported by Nuhwa et al. (2019), who screened 16 LAB isolates from edible flowers for in vitro probiotic-related traits, including tolerance to acidic and bile conditions, bile salt hydrolase (BSH) activity, and cholesterol assimilation. 11 isolates were BSH positive and all strains displayed measurable cholesterol assimilation, ranging from 9.57% to 51.69% (with the highest value recorded for isolate FM11-2), supporting the gastrointestinal resilience and metabolic potential of flower-associated LAB. While these findings are promising, further validation in in vivo settings (e.g., murine or clinical models) would be valuable to confirm their translational relevance. The use of *Caenorhabditis elegans*, a simplified eukaryotic system for studying host–microbe interactions, enabled the observation of systemic effects in nematodes fed with *Leuc. mesenteroides* C2 and *Leuc. mesenteroides* C7 isolated from carrot epidermis, including increases in longevity and enhanced stress resistance following exposure to reference pathogens (Schifano et al. 2021; Pompa et al. 2023). However, from a microbiological and applicative safety perspective, it is essential to underline the need for a preliminary safety assessment of strains intended for food use. Such analyses should include *i*) screening for haemolytic activity, *ii*) evaluation of the absence of antibiotic resistance genes, and *iii*) determination of the ability to produce biogenic amines, ideally using validated analytical techniques such as HPLC or LC-MS, in line with recommendations from the European Food Safety Authority (EFSA).

### Exopolysaccharides production and texture improvement in plant matrices

Another aspect of particular relevance is the contribution of exopolysaccharides (EPS) production to vegetable fermentation technology (Hernández-Figueroa et al. 2025). Historically, EPS production has been widely explored in the dairy context, where viscous dextrans and emulsifying compounds contribute to improving product consistency and stability (Duboc and Mollet 2001). Considering the recent evidence, the transition from dairy to plant-based fermentations necessitates a radical reevaluation of starter strain selection and design, with implications for plant-based dairy analogues. The marked differences in the physicochemical composition between animal and plant matrices, in terms of protein content, polysaccharide profile, presence of anti-nutritional factors, and fibre structure, crucially influence the metabolic activity of the strains used. It is therefore necessary to emphasise that the technological and sensory success of plant fermentations relies on identifying microorganisms that are both well-adapted and functionally suited to the specific requirements of the substrate. Concrete examples of EPS-induced texturing are increasingly reported for this kind of LAB isolation. For example, Wang et al. (2023) showed that dextran produced by *Leuc. mesenteroides* RSG7, isolated from *pepino*, improved the structural properties of sucrose-rich formulations in a skim-milk model system. Recent data highlight the existence of real microorganism-substrate co-adaptation phenomena, which influence the synthesis of key metabolites, such as EPS and aromatic compounds. As previously mentioned, edible flower isolates belonging to *Leuconostoc*, *Weissella*, *Lactococcus*, *Apilactobacillus*, and *Fructobacillus*, which were screened for their suitability in small-scale fermentations, were shown to synthesise glucans, fructans, and galactans during the fermentation of plant-based beverages. Importantly, the type and relative abundance of these polysaccharides varied across microbial species and substrates (Khiabani et al. 2024). These interactions ultimately shape the sensory quality and texture stability of plant-based fermented foods. Considering these interactions is today essential for the rational design of plant-based fermentations with high technological and nutritional value.

### Valorisation of LAB by-products and silage applications

Harnessing LAB-driven lactic acid fermentation represents an effective strategy to add value to plant-derived substrates, while reducing reliance on synthetic preservatives and minimising environmental burdens associated with chemical inputs. LAB metabolism involves interconnected biosynthetic pathways that convert matrix nutrients (e.g., carbohydrates, lipids, proteins, and selected micronutrients) into metabolites with bioprotective activity (Khubber et al. 2022). Several authors over the last decade have investigated the feasibility of exploiting selected LAB strains for the bioconservation and valorisation of agri-food by-products. For instance, nine *Lpb. plantarum* strains isolated from tropical fruits (banana, papaya, pineapple and orange) exhibited strong acidifying behaviour alongside amylolytic and cellulolytic activities. Such functional traits enabled the efficient conversion of fruit-derived by-products into lactic acid, suggesting their suitability for sustainable by-product fermentation. A parallel area of increasing interest involves the valorisation of fruit and vegetable by-products for zootechnical use. In the presence of adequate levels of water-soluble carbohydrates (WSC), it is well documented that lactic acid fermentation of forage materials can lead to silage with reduced nitrogen degradation and improved dry matter (DM) recovery (Du et al. 2021; Gao et al. 2022). This process enhances forage stability during storage and after exposure to air, thereby reducing losses and increasing animal feed intake, with a direct impact on feeding efficiency. Non-commercial or substandard fruits and vegetables represent promising substrates for the production of functional silage (Yang et al. 2016; Pereira et al. 2019). Yang et al. (2022) investigated the fermentation of non-commercial bananas using the *Lpb. plantarum* strain CG1, evaluating the feasibility of producing silage with enhanced nutritional value through co-application of tannase and sucrose. Such approaches exemplify resource-efficient models in feed biotechnology, transforming low-value agricultural residues into high-efficiency feed resources. This allows for a reduced reliance on conventional raw materials, cost-effective feed formulation, improved year-round availability of preserved forage, and more effective mitigation of seasonal feed shortages. This highlights how valorising agri-food by-products through lactic fermentation can actively contribute to integrated circular bioeconomy strategies and climate-resilient livestock models.

### Conclusions and future trends

Although the fermentative, probiotic, and antimicrobial properties of this microbial macrocategory are now well-documented, the effective translation of these traits into innovative industrial and food applications is often limited by an incomplete understanding of their interactions with vegetable matrices and the human host. The wide physiological and metabolic heterogeneity among strains requires optimising cultivation conditions and tailoring specific formulations to each substrate to promote optimal growth and maximise fermentative and functional performance. In this perspective, we outline two key research priorities: the first concerns the rational design of suitable starters with matrix-adapted specificity; the second involves the mapping of genome-environment relationships through pangenomic analyses (i.e., comparative analysis of the full gene content across different strains), gene-trait (linking phenotypic traits to specific genetic determinants) association studies, and in situ multi-omic approaches, aimed at achieving an integrated understanding of the adaptive and functional dynamics of LAB in plant ecosystems. Future research should also shift its focus from simple in vitro characterisation to the integrated analysis of in vivo models, such as rodents or zebrafish, and more comprehensive functional studies. Of course, some inherent limitations of the present review need to be pointed out, mainly related to the heterogeneity of available studies. Furthermore, the number of reported studies may be underestimated due to linguistic and coverage biases related to indexing and source retrieval criteria. Integrating ecological, omic, and technological approaches will be essential to harness plant-derived LAB for sustainable agri-food innovation.

## Data Availability

No datasets were generated or analysed during the current study.
